# Reconceptualizing cancer immunotherapy based on plant production systems

**DOI:** 10.4155/fsoa-2017-0018

**Published:** 2017-07-12

**Authors:** Kathleen Hefferon

**Affiliations:** 1 Department of Food Sciences, Cornell University, Ithaca, NY 14853 USA

**Keywords:** antibody, cancer immunotherapy, oncovirus, plant virus nanoparticle, transgenic plant

## Abstract

Plants can be used as inexpensive and facile production platforms for vaccines and other biopharmaceuticals. More recently, plant-based biologics have expanded to include cancer immunotherapy agents. The following review describes the current state of the art for plant-derived strategies to prevent or reduce cancers. The review discusses avenues taken to prevent infection by oncogenic viruses, solid tumors and lymphomas. Strategies including cancer vaccines, monoclonal antibodies and virus nanoparticles are described, and examples are provided. The review ends with a discussion of the implications of plant-based cancer immunotherapy for developing countries.

## Lay abstract

Cancer immunotherapy has made great strides over recent years. This review describes the use of plants as production systems to produce biopharmaceuticals such as vaccines and antibodies to treat a wide variety of cancers. The use of nanoparticle technology based on plant viruses as a novel strategy to target and combat cancers is also included. The review concludes with a discussion of plant production platforms and their relevance for the generation of cheap and effective cancer immunotherapies for developing countries.

Cancer is often described as a class of diseases involving the uncontrolled growth and spread of abnormal cells [1]. These cells can proliferate uncontrollably to form tumors, or else migrate by metastasizing to generate secondary tumors elsewhere in the body. Cancer is often treated by radiotherapy, chemotherapy, anticancer drugs or surgery, all expensive ventures and with a number of adverse effects [2]. Since the incidence of cancer is increasing in both developing and developed countries, the search for cost-effective new technologies that are cost effective has become of great interest. Recently, an approach known as immunotherapy, which employs the body's immune system to prevent or suppress the progression of a variety of cancers, has shown considerable promise [3]. For many years, patients with cancer were often treated by invasive procedures such as surgery followed by chemotherapy. Immunotherapy has now blossomed as an attractive alternative to conventional cancer therapies. An assortment of novel cancer-targeting antibodies that have undergone US FDA approval is now employed successfully to treat a variety of cancers, ranging from solid tumors to hematological malignancies.

Plant-derived pharmaceuticals have been under development for over 20 years. They are safe, facile to upscale, inexpensive to produce, lack refrigeration requirements and do not involve medical infrastructure to administer. One original driving force for the development of plant-made vaccines was to generate vaccines against infectious diseases that continue to cause high levels of infant mortality for resource poor countries. More recently, however, plant-made pharmaceuticals have blossomed into an industry of their own right. Today, plants have been used to produce vaccines to combat global pandemics ranging from influenza to Ebola virus. Other uses have included stockpiling vaccines against potential biological warfare agents, and for treating orphan diseases that are poorly funded.

Plants have been developed in multiple ways for biopharmaceutical production (Figure 1). Originally, the vaccine of interest was expressed stably from transgenic plants, with nuclei and chloroplasts (plastids) being the avenue of transformation. Later, transient expression technologies such as agrobacterium infiltration and plant virus expression vectors have become enticing, due to their ability to produce foreign proteins both rapidly and at high levels. For example, the Tobacco mosaic virus (TMV)-based MagnICON system is composed of a deconstructed virus genome [4]. Deconstructed expression systems are easy to manipulate and are biocontainable because the portions of the viral genome are divided into modules, which upon entry into plant cells can recombine into a fully functional replicon. Today, there are benefits and pitfalls to either approach, and decisions about which production platform to use are based on the nature and future use of the protein that is to be produced. For example, in certain instances, it may be advantageous to produce the pharmaceutical product in transgenic plants, which can be easily stored as seed and upscaled *en masse* when needed. In other cases, virus-like particles (VLPs) may be useful for rapidly producing a vaccine for an infectious disease with a constantly changing epitope, such as pandemic influenza [4]. Plants have been generated to produce vaccines in the form of VLPs, subunit or full protein vaccines, more complex biologicals such as monoclonal antibodies, and more recently, plant virus nanoparticles have been designed as immunotherapies against a variety of diseases [5,6].

**Figure 1.**
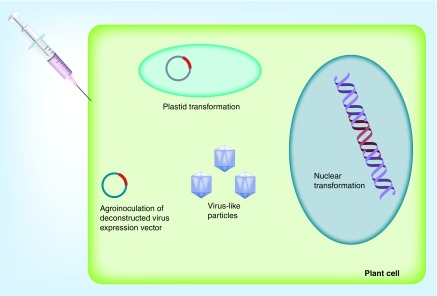
**Different types of biopharmaceutical expression strategies utilizing plant-based production systems.**

As plant expression systems become more sophisticated, they are being incorporated more and more as immunotherapy strategies for a variety of cancers. This review covers some of the most recent developments with respect to plant-derived pharmaceuticals that have been developed to fight cancer. The review includes an examination of potential vaccines to combat oncolytic viruses, autologous vaccines for lymphomas and solid tumors, and monoclonal antibodies to target cancers. The development of plant nanoparticles for immunotherapy is described. The review concludes with a discussion of possible implications of these technologies for the developing world.

## Plant-derived vaccines and immunotherapies for oncogenic viruses

### Hepatitis B virus

Hepatitis B virus (HBV) is a disease of paramount importance, with a third of the world's population carrying the disease burden. HBV is a causative agent of chronic liver disease, including cirrhosis and hepatocellular carcinoma. About 600,000 people die around the globe each day from virus infection, and hepatitis B is responsible for about 66% of hepatocellular carcinoma cases worldwide [7]. Infected individuals generate large amounts of HBV surface antigen (HBsAg), which can assemble as strongly immunogenic VLPs lacking nucleic acid and thus noninfectious in the bloodstream. These 22-nm VLPs have been a source of vaccine design for many years. In developing countries, however, where HBV infection continues to predominate, accessibility to vaccines against HBV has been unfeasible due to high cost and lack of medical infrastructure. As a result, from the early onset of plant-based vaccine development, much attention has been placed on the development of an inexpensive, ‘needle-free’ plant-based vaccine against HBV that is stable at room temperature for prolonged periods of time and is both intrinsically safe and efficacious [8,9]. Unfortunately, all of these efforts have focused on creating vaccines to prevent HBV infection and do not reflect efforts to control the onset of liver cancer in chronically infected individuals.

Originally, transgenic plants were used to generate vaccines to HBV. Crude plant extracts were tested on animal models such as mice [10]. Later, transgenic potato tubers expressing HBV were fed to human volunteers as a proof of concept that oral consumption of a plant-based vaccine could elicit a strong immune response [11]. Since then, many approaches have been taken for the development, processing and manufacturing of plant-based vaccines to HBV in plant varieties such as lettuce, tomato, maize and rice [12–20].

For example, Hayden *et al*. recently used maize germ wafers expressing HBsAg as part of a prime-boost vaccination strategy [21]. Mice were injected with a primary dose of Recombivax and later provided with an oral boost of HBsAg wafers. Mice treated with this vaccine regimen demonstrated mucosal IgA increased titers and long-term memory over 1-year postvaccination, indicating that this strategy may provide additional protection to a conventional vaccine regimen. As another example, Pniewski *et al*. used micropropagation techniques to develop transgenic lettuce lines expressing high and uniform levels of HBsAg [17]. The authors hope to use this strategy as an advance toward the manufacture of a standardized oral vaccine for HBV.

Although a plant-based HBV vaccine has been under development for two decades now, a commercial vaccine based on this technology has yet to be realized. One of the principal reasons for this is the low expression levels of HBV vaccine in plants. Efforts to improve vaccine yields as well as to better stimulate the immune response are currently underway.

## Plant-derived vaccine against hepatitis C virus

Hepatitis C virus (HCV) is another major cause of acute and chronic hepatitis and liver cancer. Today there are over 185 million cases of HCV worldwide [22]. Vaccine development for HCV has been challenging due to difficulties growing the virus in tissue culture. Initially, TMV was used as a vector to express potential neutralizing epitopes of HCV that had been fused to the strong immunogen subunit B of cholera toxin [23]. Cucumber mosaic virus (CMV) has also been used as an expression vector system to express HCV hypervariable region (HVR1) epitopes from its coat protein. Serum from HCV-infected patients cross-reacted with plant extracts taken from plants infected with the recombinant virus [24]. Piazzolla *et al*. and Nuzzaci *et al*. further demonstrated that plant extracts containing the CMV-HVR1 vaccine could elicit an increase in IFN-γ and cytokines in lymphocytes [25,26]. Nuzzaci *et al*. later showed that recombinant CMV infecting lettuce that was fed to rabbits could elicit an HCV-specific immune response [27].

The HRV1 epitope found on the envelope protein of HCV has also been expressed in tobacco plants using recombinant vectors based on Alfalfa mosaic virus and TMV [28]. Uhde-Holzem *et al*. used potato virus X (PVX) to express HVR1 as a fusion protein including the ribosomal skip (2A) sequence [29]. Mice immunized parenterally elicited an IgG response and sera from chronically infected HCV patients reacted positively to the plant-derived vaccine. Papaya mosaic potexvirus (PapMV) has also been used to express the envelope protein of HCV. PapMV VLPs which express this epitope in mice generated a long-lasting humoral antibody response [30].

Mohammadzadeh *et al*. fused specific immunogenic epitopes derived from HCV core antigen to HBsAg and expressed this chimeric construct from the second coat protein promoter of a PVX vector [31]. The construct was co-infiltrated into tobacco leaves along with suppressor of gene silencing P19, derived from tomato bushy stunt virus.

Natilla *et al*. expressed HCV core antigen in transgenic tobacco plants and were able to recognize the HCV in infected human serum [24]. HCV core protein has also been expressed in transgenic *Brassica napus* seeds [32]. In this study, the core antigen was plant optimized and expressed under the control of the seed-specific promoter, fatty acid elongase 1.

## Plant-derived vaccines to treat cervical cancer

Cervical cancer is the second most common cancer in women and is attributable to human papillomavirus (HPV). HPV is sexually transmitted and represents a significant economic and health burden in developing countries. HPV is a small, double-stranded DNA virus and infects both squamous and cutaneous epithelial cells in humans. HPV genotypes 16 and 18 are the most prevalent; together they are responsible for over 80% of cervical cancer cases found around the world. The capsid of HPV is icosahedral and is composed of the structural late proteins L1 and L2. Early proteins E6 and E7 are responsible for oncogenicity of the virus. The virus capsid proteins can self-assemble in the absence of viral DNA to form empty VLPs. HPV VLPs are noninfectious and highly immunogenic, and thus have been a source of vaccine development.

Currently, two vaccines in the form of VLPs, Gardasil (Merck, NJ, USA) and Cervarix (GlaxoSmithKline, Brentford, UK), are commercially available; however, they protect against a limited number of HPV genotypes, and high production costs limit their accessibility for those who reside in developing countries. The rapid scalability and facile production of plants as production platforms for human biologics have led to the concerted efforts of a number of research groups to focus on a plant-derived vaccine to HPV as a preventative measure against cervical cancer. Extensive studies based on plant-derived vaccines which specifically target HPV and cervical cancer can be found in the following review [33] and its references: transgenic and agrobacterium-infected plants [34–37], plant virus vectors [38–41] and plastids [42–44]. While earlier research reports described relatively low expression of HPV VLPs in plants, the ongoing evolution of plant expression systems has vastly increased levels over time. This section serves to highlight a few of the more recent developments with respect to the use of plants to block cervical cancers that result from HPV infection.

Plant virus expression vectors have been utilized to produce HPV vaccines. For example, Zahin *et al*. have optimized methods to express VLPs based on HPV16 L1 protein using the TMV-deconstructed vector MagnICON expression system from tobacco plants [45]. One construct that appeared to be expressed most effectively in producing VLPs contained the full length open reading frame of L1, fused to a chloroplast transit peptide. The authors predict from their study that this chloroplast-targeted transient expression technique could feasibly generate a cervical cancer vaccine based on HPV16 that could be both inexpensive and efficacious to produce *en masse* for low-income countries.

Lamprecht and co-workers in South Africa have utilized a plant geminivirus expression system to express constructs containing HPV L1 and L2 capsid proteins along with a secreted embryonic alkaline phosphatase reporter construct to express and assemble HPV pseudovirions (PsV) in plants for the first time [46]. The reporter construct was expressed in the form of a circular dsDNA replicon, which could then be encapsidated by L1 and L2 into PsV in tobacco plants. The plant-based PsV could be easily purified and used to identify neutralizing antisera collected from patients in a manner that is far more cost effective than currently available mammalian systems.

Furthermore, Lössl and co-workers have fused L1 of HPV16 to the affinity tag glutathione-S-transferase (GST) and expressed pentameric capsomers of these fusion proteins in tobacco chloroplasts [47]. Pentameric capsomers, like VLPs, are highly immunogenic. L1 expressed in transplastomic lines retained their immunogenic properties, as determined by antigen capture ELISA, and plants expressing GST-L1 appeared identical to wild-type plants in both morphology and fertility.

In addition to the use of the structural proteins L1 and L2 to generate vaccines based on VLPs, plant-based immunotherapies have also been pursued for patients who are persistently infected with HPV. In these cases, the strategy has been to induce an immune response to HPV oncoproteins E6 and E7. Franconi *et al*. have used the potexvirus PVX to express E7 of HPV and effectively block tumor growth in mice [48]. The same group also fused a modified version of E7 to a plant lichenase protein, to function as an adjuvant and improve the immune response. This fusion construct was expressed from a ‘launch vector’ based on TMV and successfully prevented tumor growth. The vaccine was able to cure established tumors in both nonorthotopic and orthotopic mouse models, respectively, further validating the feasibility of plant-based immunotherapy for HPV-infected patients [49].

## Autologous (patient-specific) vaccines & personalized medicines

Non-Hodgkin's lymphoma (NHL) is the most common hematologic malignancy in the USA, with an estimated 70,000 new cases in 2014 alone. Of these, approximately one-quarter of cases are follicular B-cell lymphoma, and patients with this disease are treated by chemotherapy, radiation, passive antibody therapy and, more recently, active immunotherapy [50]. Since malignant B cells each express a unique cell surface idiotype that is specific to that individual, patients can be vaccinated using their own idiotype produced from a variety of expression systems, ranging from bacterial, insect or mammalian cells to traditional hybridomas. As a result, immunotherapy for every patient is highly specific, expensive and timely. Plants were considered to be a possible expression platform for personalized medicine to block NHL due to their flexibility, low cost, rapid and high yielding production, and quality compliance.

Two different constructs have been used as experimental NHL cancer vaccines; one was a single-chain Fv antibody fragment (scFv) subunit comprised of only the tumor-specific idiotope and fused to a TMV-based expression system [51]. The second construct consisted of full length idiotype IgG molecules that were expressed in deconstructed MagnICON vectors as heavy and light chains, which assembled into full immunoglobulins in the plant [52]. Both vaccine constructs have successfully undergone FDA qualified Phase I clinical trials with a number of lymphoma patients and were demonstrated to be safe, and elicit few adverse effects. Disease progression was assessed for up to 3 years postvaccination regimen for each lymphoma patient. Parenteral administration of these vaccines resulted in immune responses specific to individual tumor idiotypes for the vast majority of patients, and not to other extraneous plant antigens that were retained during the purification process. The number of patients who mounted immune responses (82% positive humoral and/or cellular) was comparable with results of earlier clinical trials using follicular lymphoma idiotype vaccines that have been generated using other production platforms. Furthermore, vaccine manufacture was extremely rapid, taking less than 3 months to obtain a completed vaccine based upon FDA cGMP guidance from an initial biopsy [53,54].

Jobsri *et al*. used plant virus-based nanoparticles containing idiotypic tumor antigens as a conjugate vaccine in order to elicit an immune response against murine B-cell lymphoma [55]. In this case, the viral ssRNA acts as an adjuvant via TLR7 engagement to stimulate IFN-α secretion. The authors coupled the idiotype of individual patients as an antigen to the surface of potexvirus PVX, which is known to elicit a robust T-cell response.

## Plant-derived vaccines against solid tumors

Plant-derived vaccines have also been explored as a potential immunotherapy against solid tumors. For example, the MagnICON plant virus expression system has been constructed to display an epitope to Her2 that is responsible for eliciting a strong immune response against breast cancer. This Her2-targeting vaccine required conjugation to a foreign immunogenic carrier, fragment C of tetanus toxin, to be effective. The vaccine was proven to be successful in a mouse model [56]. Alternatively, Esfandiari *et al*. used PVX nanoparticles to transport monoclonal antibodies of herceptin (Trastuzumab), as a targeted therapy in (Her2+) for breast cancer patients [57]. These PVX-based nanofilaments were able to induce apoptosis in breast cancer cell lines.

Matić *et al*. used the Cowpea mosaic virus (CPMV) expression vector pEAG-HT to express the extracellular domain of the rat ErbB2 in *Nicotiana* plants [58]. ErbB2 belongs to the EGF-related proteins (ErbB) of receptor tyrosine kinases, and its aberrant expression can lead to a variety of cancers. Mice injected with plant extracts expressing ErbB2 elicited immune responses and potent antitumor activity, demonstrating the effectiveness of this plant-based immunotherapy approach.

Similarly, Pinkhasov *et al*. expressed a highly immunogenic epitope from the human epithelial mucin MUC1, fused to *Escherichia coli* heat-labile enterotoxin B subunit (LT-B) as an adjuvant in plants [59]. MUC1 is a transmembrane protein that is overexpressed and aberrantly glycosylated in the majority of human breast cancers. The MagnICON system was used as a production platform, and antibodies against MUC1 were observed; these were as functional as conventionally made antibodies from mammalian cell culture.

Other research groups have developed plant-based immunotherapies for colorectal cancer. For example, Ahn *et al*. fused the colorectal cancer antigen GA733 to an Fc antibody fragment that contains the endoplasmic reticulum retention motif KDEL in transgenic plants [60]. Endoplasmic reticulum retention facilitated accumulation of the antigen with the correct oligomannose glycosylation motifs and resulted in a vaccine that was comparable in immunogenicity to its mammalian-derived counterpart [61].

Prostate cancer is the sixth leading cause of cancer for men in the world, and is associated with the loss of ability of prostate epithelial cells to undergo apoptosis. Par-4 protein can cause tumor regression in animal models by promoting apoptosis of cancer cells. *Par-4* is often found to be downregulated or has suffered deleterious mutations in cancer patients. The SAC domain of Par-4, highly conserved among humans, mice and rats, represents the domain responsible for inducing apoptosis, and has been utilized for anticancer regimens to induce tumor suppression. SAC of Par-4 has been fused to green fluorescent protein and expressed by [62,63] in transgenic tobacco plants. Anticancer effects of plant-derived SAC were confirmed by a variety of cell proliferation assays. Tumor progression was also examined in Copenhagen rats. The results of this study reveal that plant-derived SAC has a cytotoxic effect on cancer cell lines, can induce apoptosis of prostate cancer cells and reduce tumor progression in an animal model.

## Production of plant-derived monoclonal antibodies to combat cancer

Besides NHL, plants have successfully been developed to produce monoclonal antibodies for cancer immunotherapy [64]. Monoclonal antibodies have been shown to trigger a localized immune response against tumors through the activation of macrophages, natural killer cells and the complement system, resulting in antibody-dependent cellular cytotoxicity and complement-dependent cytotoxicity [65–67]. However, monoclonal antibody therapy has been difficult to implement due to accessibility with respect to cost and scalability, as well as the potential for contamination of mammalian expression systems with human pathogens. The need for post-translational modification also inhibits the use of bacterial expression systems for the production of monoclonal antibodies for therapeutic use.

The generation of monoclonal antibodies in plants for cancer immunotherapy is not a recent accomplishment; rather, it has been in the pipeline throughout the history of plant-derived pharmaceutical development [68–73].

## Plant virus nanoparticles to block tumor progression

Nanoparticle technology has been applied in interesting ways to cancer immunotherapy. Synthetic nanoparticles are able to elicit a localized immune response against cancer cells [74]. Because plant viruses are able to self-assemble into organized structures, they can be utilized as nanoparticles which are stable, nontoxic and biodegradable [75]. Plant viruses are attractive because they do not cause disease in humans, and are now used in targeted imaging and therapy. The external surface of rod-shaped plant virus particles such as potexviruses can become functionalized through modification to target delivery to cancer cells. Icosahedral plant viruses such as CPMV can be modified to carry cargo molecules such as drugs within their internal cavity. In this way, plant viruses can be used for diagnosis as well as for immunotherapy [74,75].

Plant viruses have also been developed for their unique immunostimulatory properties, as well as for their use in drug design and the medical imaging of tumors. Plant virus nanoparticles have been utilized to block the progression of several types of cancer, further supporting their potential role in medicine. Different plant virus nanoparticles are distributed differentially throughout the body and can target different tissue types [76]. Nanoparticle morphology and molecular composition also play a role in determining the type of immune response that is elicited [77,78].

PVX, for example, contains external lysine residues on the exterior loop of the capsid protein which can be functionalized to adhere to conjugates such as drugs or imaging reagents. Stability of these virus nanoparticle conjugates could be improved by PEGylation. Shukla *et al*. examined the tumor homing efficiency of PVX in different cancer animal models, and found that PVX nanoparticles could accumulate in the center of solid tumors, suggesting that elongated nanoparticles such as PVX have an easier time penetrating deeper through the tumor to its core [76,79].

The potexvirus PapMV also contains immunostimulatory properties. The unaltered virus can elicit an IFN-α-dependent response and, when administered intratumorally, can slow down melanoma progression and prolong survival in animal models [77,80]. The virus elicits an increase in inflammatory cytokine production and infiltration of CD8^+^ T cells, in addition to a decrease in myeloid-derived suppressor cells [81–83]. PapMV undergoes rapid endocytosis by antigen-presenting cells, where its single-stranded RNA is recognized by TLR7, resulting in IFN-α induction, an increase in MHC-1 surface expression and CD8^+^ T-cell proliferation. This leads to a blockage of tumor progression in mouse models. Mice who were treated systemically with PapMV followed by B16-OVA cells 6 h later exhibited a large reduction in tumor nodule number when compared with untreated mice used as controls [84].

The icosahedral CPMV can bind to vimentin displayed on the surface of HeLa cells and can be internalized by endocytosis [85,86]. CPMV can also be internalized by antigen-presenting cells and directly stimulate the immune system to combat various cancers [87]. CPMV capsid proteins can be driven to self-assemble in the absence of RNA to form empty noninfectious VLPs, which when applied to a tumor can alter the surrounding microenvironment to potentiate tumor immunity [88]. This highly localized immunization strategy lessens the chance of patients encountering an adverse response.

CPMV nanoparticles have been shown to act as effective immunotherapies for lung melanoma and other cancers in a mouse model, and can induce innate immune-cell-mediated antitumor responses [89]. CPMV nanoparticles provided to bone marrow cell cultures also increased proinflammatory cytokine levels. Furthermore, inhalation of CPMV nanoparticles dramatically increased neutrophil populations within a day after administration, and weekly injection with the nanoparticles reduced tumor burden, suggesting that neutrophils are required for the CPMV-directed antitumor effect. The immunoregulatory nature of CPMV nanoparticles was successful for a variety of tumors, ranging from ovarian and breast tumors to colon tumor models, and can delay tumor progression as early as 3 days postinjection. Tomato bushy stunt virus is another icosahedral virus that has been designed to encapsulate small molecules and display peptides [90].

TMV can also be made to be tumor specific using a tumor-homing peptide (such as cRGD, which is selective for ligands growing on growing tumors) that is functionalized to the surface of the virus [91]. TMV displaying cRGD can be rapidly internalized into tumor cells. Chemotherapeutic drugs such as doxorubicin can also be conjugated to TMV and released during endocytosis within cancer cells.

Plant virus nanoparticles have been used as tissue-targeting imaging vehicles as well [92]. The use of plant virus nanoparticles for imaging depends on the use of fluorochromes and specialized scanning microscopy formats such as ‘two-photon laser scanning microscopy’ [93]. Virus nanoparticles based on PVX and CPMV have been engineered to incorporate fluorescent dyes and are used as highly specific cancer imaging reagents through conjugation with PEG polymers and a number of different targeting ligands [94]. For example, CPMV has been shown to home in on tumors in an HT-29 colon cancer mouse model [95].

## Conclusion & future perspective: implications for the developing world

Plant-made biopharmaceuticals to fight cancer are beginning to blossom as a field in their own right. As the world proceeds to alleviate many of the most deadly infectious diseases known to mankind, attention has shifted toward resolving chronic health conditions such as cancer. This review has included the use of plant-made vaccines to combat oncolytic viruses that are of concern to resource poor countries, such as HBV, HCV and HPV. Efforts to decrease the incidence of infection by these viruses will make a huge difference to the quality of life for hundreds of millions. The generation of vaccines and monoclonal antibodies, in a manner that requires little processing or medical infrastructure, strongly supports the exploration of plant-derived biologics as a means to assist developing countries. The recent discovery of plant virus nanoparticles to target and penetrate solid tumors is particularly exciting [96,97]. Nanoparticles based on plant viruses can be readily produced with drugs functionalized to their surfaces, thus enabling them to more easily home in and block tumor progression. Their low cost, ease of *en masse* generation and low toxicity make plant nanoparticles ideal for a resource-poor setting [98]. Nanoparticles have demonstrated clear efficacy on many different cancer models.

As an alternative, it is also possible that plant-made pharmaceutical research and development could become ‘homegrown’ in developing countries. Equipping them with constructs, transgenic seeds and freedom to operate makes the production of plant-made pharmaceuticals a feasible direction for a country to explore. However, improvements in manufacturing infrastructure, financial support and the maturation of a regulatory framework surrounding plant-derived pharmaceuticals are all essential for their commercialization in the developing world. Since much of the source of plant-based biologic research and development has come from academia, the movement from research and development to the corporate arena will take a considerable effort to say the least. Nonetheless, attractive features such as lower production costs, fewer purification steps, and high levels of safety and efficacy make plant-derived pharmaceuticals a platform technology that suits the needs of developing world markets. It is hoped that for resource-poor nations, plant-made vaccines and biologics designed to defeat cancer will successfully make the move from concept to reality (Table 1).

**Table 1.** **Select examples of plant production systems used for cancer immunotherapy.**

**Malignancy**	**Antigen/plant system used**	**Ref.**
Hepatitis B virus induced hepatocellular carcinoma	HBsAg expressed in transgenic plants	[11]
Hepatitis C virus induced hepatocellular carcinoma	E7 protein expressed in chloroplasts	[34]
Non-Hodgkin's lymphoma	Full IgG expressed in TMV-based expression system	[52]
Breast cancer	PVX nanoparticles expressing HER2 epitope	[57]
Solid tumors	PapMV nanoparticles	[80]
Lung melanoma	CPMV nanoparticles	[89]
Solid tumors	TMV nanoparticles displaying cRGD	[91]

CPMV: Cowpea mosaic virus; HBsAg: Hepatitis B virus surface antigen; PapMV: Papaya mosaic potexvirus; PVX: Potatovirus X; TMV: Tobacco mosaic virus.

**Executive summary**

- Cancer immunotherapy has gained momentum as an effective and noninvasive way to treat cancer patients.
- Plant production platforms have been developed to produce cancer vaccines and monoclonal antibodies that are directed against a variety of cancers, ranging from oncolytic virus-derived cancers to breast cancers.
- Plant viruses have been engineered as nanoparticles to target solid tumors and induce an immune response. Some plant viruses can carry ‘payloads’ of anticancer drugs.
- Inexpensive, safe and efficacious biopharmaceuticals produced using plant production systems could have great implications for cancer patients residing in resource-poor countries.
